# Transcriptomic and Proteomic Analysis of *Arion vulgaris*—Proteins for Probably Successful Survival Strategies?

**DOI:** 10.1371/journal.pone.0150614

**Published:** 2016-03-17

**Authors:** Tanja Bulat, Roman Smidak, Fernando J. Sialana, Gangsoo Jung, Thomas Rattei, Martin Bilban, Helmut Sattmann, Gert Lubec, Jana Aradska

**Affiliations:** 1 Department of Pediatrics, Medical University of Vienna, Vienna, Austria; 2 Division of Computational System Biology, Department of Microbiology and Ecosystem Science, University of Vienna, Vienna, Austria; 3 Department of Laboratory Medicine and Core Facility Genomics, Medical University of Vienna, Vienna, Austria; 4 Third Zoological Department, Museum of Natural History Vienna, Vienna, Austria; 5 Department of Pharmaceutical Chemistry, University of Vienna, Vienna, Austria; Institute of Oceanology, Chinese Academy of Sciences, CHINA

## Abstract

The Spanish slug, *Arion vulgaris*, is considered one of the hundred most invasive species in Central Europe. The immense and very successful adaptation and spreading of *A*. *vulgaris* suggest that it developed highly effective mechanisms to deal with infections and natural predators. Current transcriptomic and proteomic studies on gastropods have been restricted mainly to marine and freshwater gastropods. No transcriptomic or proteomic study on *A*. *vulgaris* has been carried out so far, and in the current study, the first transcriptomic database from adult specimen of *A*. *vulgaris* is reported. To facilitate and enable proteomics in this non-model organism, a mRNA-derived protein database was constructed for protein identification. A gel-based proteomic approach was used to obtain the first generation of a comprehensive slug mantle proteome. A total of 2128 proteins were unambiguously identified; 48 proteins represent novel proteins with no significant homology in NCBI non-redundant database. Combined transcriptomic and proteomic analysis revealed an extensive repertoire of novel proteins with a role in innate immunity including many associated pattern recognition, effector proteins and cytokine-like proteins. The number and diversity in gene families encoding lectins point to a complex defense system, probably as a result of adaptation to a pathogen-rich environment. These results are providing a fundamental and important resource for subsequent studies on molluscs as well as for putative antimicrobial compounds for drug discovery and biomedical applications.

## Introduction

*Arion vulgaris* as the only terrestrial gastropod, is considered among the 100 worst invasive species in Europe. The spread of invasive species, including slugs, is an increasing problem worldwide with an important economical, ecological, health and social impact. The slug *A*. *vulgaris* is considered a serious pest, both in agriculture and private gardens. Moreover *A*. *vulgaris* may act as a vector for some pathogenic bacteria, like *Listeria monocytogenes* [1], *Clostridium botulinum* [2],[3] and host species for some parasites, as *Angiostrongylus vasorum* that can cause serious and potentially fatal disease in dogs and other canids [4]. *A*. *vulgaris* belongs to a species complex *Arion ater* that can only be distinguished by dissecting their reproductive organs.

Currently, transcriptomic studies on molluscs have been restricted to an ecological framework in the marine and fresh water gastropods. However, no transcriptomic or proteomic study has been carried out so far in *A*. *vulgaris*. Next-generation sequencing has offered a powerful and cost-efficient technique for the generation of transcriptomic datasets in non-model species using diverse platforms such as the Illumina HiSeq, Roche 454, Pacific Biosystems, and Applied Biosystems SOLiD. Several non-model organisms have been characterized by transcriptome sequencing [5–7], which has provided a better understanding of these species.

Since mRNA expression does not necessarily reflect changes at the protein level, complementary proteomic studies can yield more comprehensive biological insight. Mass spectrometry analysis allows large proteome surveys and comparative analysis, where quantities of hundreds or thousands of proteins can be compared between various conditions. However, the lack of a genomic resource for these animals is the major reasons for restricting proteomic applications.

In the current study, the Illumina HiSeq 2000 platform has been used to generate an *A*. *vulgaris* transcriptome-based protein database. The transcriptome data generated in this study provide a first comprehensive and valuable genomic resource for future research on this slug. With the objective to obtain insight into the slug proteome gel-based proteomic, analysis from the *A*. *vulgaris* mantle was performed.

## Methods

### Collection of Arion vulgaris

*A*. *vulgaris* specimens were collected in May 2014 in a location (48° 19′ 40″ N, 16° 12′ 34″ E) in Woerdern, Austria. A total of 15 specimens ranging from 7 to 8 g were collected. Species identification was carried out with the assistance of the Zoological Department of Museum of Natural History in Vienna (H.S.). According to national law no permission is required to collect and use invertebrates.

### Transcriptome sequencing and assembly

Isolation of RNA was performed with the RNeasy kit (Qiagen, Hilden, Germany). Following removal of the gut, two specimens of *Arion vulgaris* were immersed in liquid nitrogen and pulverized with the help of a mortar and pestle. Pooled total RNA was subjected to RNA-Seq following the mRNA sequencing protocol provided by New England Biolabs (NEBNext kit, NEB, Frankfurt, Germany). The first step in the workflow involved purifying the poly(A)-containing mRNA molecules using two rounds of poly(T)-oligo–attached magnetic beads. After purification, mRNAs were fragmented into small pieces using divalent cations under elevated temperature. The cleaved RNA fragments were copied into first-strand complementary DNA (cDNA) using reverse transcriptase and random primers. This was followed by second-strand cDNA synthesis using DNA polymerase I and RNase H (NEB, Frankfurt, Germany). These cDNA fragments subsequently went through an end-repair process, the addition of a single A base and ligation of the adaptors. The products were then purified and enriched with 15 cycles of PCR (with a size distribution of ~200–250 bp) to create the final cDNA library. Finally, the adaptor-ligated DNA was sequenced for 100 cycles on a HiSeq2000 sequencing system (Illumna, San Diego, USA) in a paired-end manner according to the manufacturer's instructions. The generated reads were filtered and trimmed by prinseq-lite using parameters trim_qual_right 30, min_qual_mean 30, min_len 70, trim_tail_right 6, trim_tail_left 6, and rRNA sequences were removed using SortMeRNA 1.99 [8]. Digital normalization (maximal coverage 30) and transcript assembly were performed using Trinity pipeline (release 2013-02-25, [9]).

### Transcriptome annotation

Protein-coding sequences were identified in the assembly using the TransDecoder tool and PFAM version 27.0. Sequence similarities were obtained by blastx searches (cut-off E-value 1E-6) of the contigs and predicted CDS, respectively, against the NCBI non-redundant protein database (version July 2014). The assembled transcripts were further annotated with GO terms, PFAM protein domains and Enzyme Commision (EC) numbers using web platform FastAnnotator [10] with the default search parameters. Duplicates and substrings were removed from the translated sequence dataset and resulting protein sequences were post-assembled using an in-house script to reduce redundancy. Final assembly containing 47,451 non-redundant protein sequences were used as an expression dataset for MS protein identification.

### Protein extraction

Individuals of *A*. *vulgaris* were frozen immediately after harvest in liquid nitrogen and stored at -80°. Mantles were dissected directly before protein extraction procedure and homogenized in urea buffer (7 M urea, 2 M thiourea, 4% CHAPS, 2 M Tris (pH 8.8), 0.5% Carrier (3–11 pH), 1% DTT, 1 mM EDTA, 1 mM PMSF, Protease Inhibitor C). Protein samples were cleaned using a 2-DE clean up kit (BioRad, Hercules, CA, USA) and protein concentration was estimated by the Pierce 660 kit (ThermoScientific, Rockford, USA).

### Two dimensional gel electrophoresis (2-DE) and nano-LC-ESI-MS/MS analysis

2-DE was performed as reported previously with minor modifications [11–13]. 750 μg of proteins were loaded on 18 cm 3–11 linear IPG strips (GE Healthcare). IEF was performed in a IPGphore electrophoretic system (GE Healthcare, Uppsala, Sweden) using a protocol with gradually increasing voltage from 200 to 8,000 V at 4 V/min. Prior to second dimension electrophoresis, IPG strips were incubated in 10 ml of equilibration buffer (50 mM Tris-HCl, pH 8.8, 6 M urea, 30% glycerol, 2% SDS and 0.002% bromophenol blue) containing 1% DTT for 15 min with gentle shaking followed by incubation in 10 mL of equilibration buffer with 4% iodoacetamide. The second-dimensional separation was performed on 10–16% gradient SDS-PAGE gels. After fixation for 4 h in 50% methanol and 10% acetic acid, gels were stained overnight with the colloidal coomassie blue (Novex life technologies, Invitrogen, CA). Molecular masses were determined by comparison with precision protein standard markers (#1610373, BioRad, Hercules, CA, USA) spanning the 10 to 250 kDa molecular weight range. All spots were excised from 2-DE gels and digested with trypsin and/or chymotrypsin. The gel pieces were cut into small pieces and washed with 50% ACN in 10mM ammonium bicarbonate for 20 min with vortexing and then 20 min with 10mM ammonium bicarbonate. These two steps were repeated until the gel was completely destained. 100% ACN was added for 10min and gel pieces were dried completely using a SpeedVac concentrator for 30 min at 30°C. Cysteine residues were reduced with 10 mM DTT at 56°C for 30 min and alkylated with 55 mM iodoacetamide for 45 min at 22°C. After washing with 10 mM ammonium bicarbonate and dehydratation with 100% ACN, proteins were digested with 12.5 ng/μL trypsin (Promega, Mannheim, Germany) solution buffered in 10 mM ammonium bicarbonate for 16 h (overnight) at 37°C. The supernatant was transferred to new LoBind 0.5 mL tubes and peptides were extracted with 1% formic acid and then with 15% ACN/0.1% formic acid [14]. 20 uL of extracted peptides were analyzed by nano-LC-ESI-(CID/ETD)-MS/MS essentially as described previously [14]. The HPLC used was an Ultimate 3000 system (Dionex Corporation, Sunnyvale, CA) equipped with a PepMap100 C-18 trap column (300 μm× 5 mm) and PepMap100 C-18 analytical column (75 μm× 250 mm). The gradient was (A = 0.1% formic acid in water, B = 0.08% formic acid in ACN) 4–30% B from 0 to 105 min, 80% B from 105 to 110 min, 4% B from 110 to 125 min. The Amazon speed ETD (Bruker Daltonics, Bremen, Germany) was used to record peptide spectra over the mass range of m/z 400–1,400, and MS/MS spectra in information-dependent data acquisition over the mass range of m/z 100–2,800. Repeatedly, MS spectra were recorded followed by three data-dependent CID MS/MS spectra. An active exclusion of 0.4 min after two spectra was used to detect low abundant peptides. The voltage between ion spray tip and spray shield was set to 1,400 V. Drying nitrogen gas was heated to 150°C and the flow rate was 3 L/min. The collision energy was set automatically according to the mass and charge state of the peptides chosen for fragmentation. Multiple charged peptides were chosen for MS/MS experiments due to their good fragmentation characteristics. MS/MS spectra were interpreted and peak lists were generated by Data Analysis 4.1 (Bruker Daltonics, Bremen, Germany).

### One dimensional (1-D) SDS-PAGE and LC-MS/MS

Although 2-DE followed by LC-MS/MS is a proven method for protein separation and identification, it suffers from poor sensitivity, poor representation of very acidic or basic proteins and low solubility of hydrophobic proteins. Therefore, we also separated proteins by 1-D SDS-PAGE coupled with high resolution LC−MS/MS. 50 μg of extracted proteins were mixed with Laemmli buffer (150 mM Tris HCl pH 6.8, 300 mM DTT, 6% SDS, 0.3% bromophenol blue, 30% glycerol) at a 1:3 volume ratio. Proteins in each sample were separated by 10% SDS-PAGE and stained by Blue silver [15]. After destaining with Milli-Q water, gels were cut into 12 slices with proteins of different mass. Proteins underwent in-gel trypsin digestion as above and were analyzed using a LTQ-Orbitrap Velos (ThermoFisher Scientific, Waltham, MA, USA) coupled with nano-LC (Dionex Ultimate 3000) as described previously [16].

### Database search

Data generated from Amazon speed ETD for each of the 2-DE protein spots were analyzed by searching the mRNA-derived *Arion vulgaris* database (47,451 sequences) including 115 commonly observed contaminants with Mascot Search enigma (version 2.4) using the Mascot Daemon interface (Matrix Science, London, UK). Detailed search criteria were used as follows; enzyme: trypsin or chymotrypsin with a maximum of two missing cleavage sites; search mode: MS/MS ion search with decoy database search included; fixed modification: carbamidomethylation (C); variable modification: oxidation (M); search mode: MS/MS ion search with decoy database search included; peptide mass tolerance 0.35 Da; MS/MS mass tolerance ± 0.35 Da.

For the mass spectrometry data generated from the LTQ Orbitrap Velos, the MS raw files were first processed with Proteome Discoverer 1.4 (ThermoFisher Scientific, IL, USA) to generate separate Mascot generic files. The database search was performed using Mascot version 2.4 (MatrixScience, London, UK) against the combined database as mentioned above. The search criteria were 10 ppm for precursor and 0.5 Da for fragments; search mode: MS/MS ion search with decoy database search included; fixed modification: carbamidomethyl (cysteine); variable modification: oxidation (methionine).

Peptide identifications were filtered to a < 1% false discovery rate (FDR) using the target-decoy strategy [17]. Acceptance parameter for protein identification was a minimum of one unique peptide and two distinct peptides.

## Results

### Transcriptome of whole body

**Fig 1:**
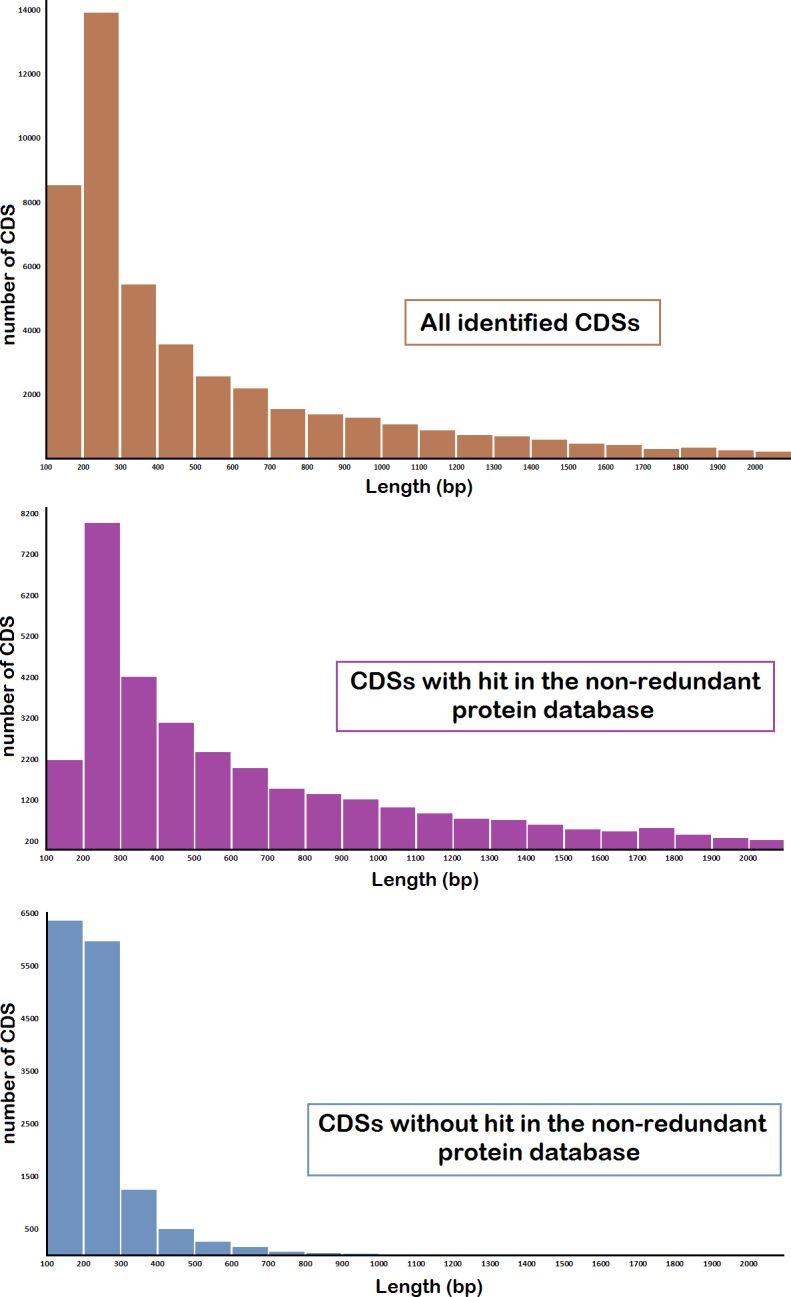
Protein-coding sequences (CDS) distribution showing the majority of sequences in the range from 200 to 300 bp.

**Fig 2:**
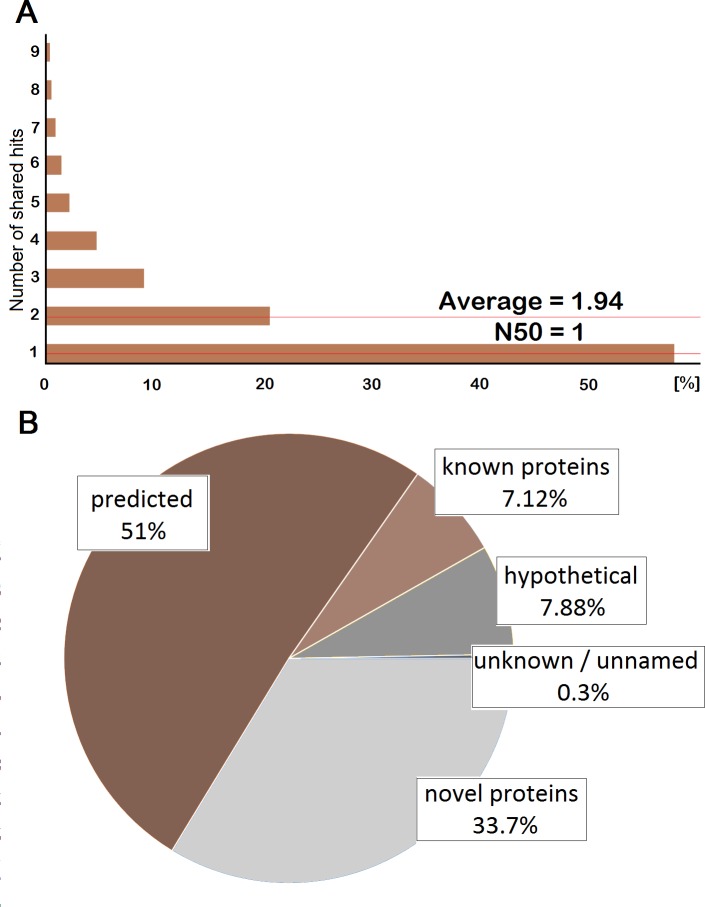
Characteristics of sequences from *A*. *vulgaris* transcriptome. (a) Distribution of shared top-hits. (b) Classification of top-hits in the current transcriptome.

The slug-specific database was constructed using a RNAseq and *de novo* assembly strategy. A cDNA library from the adult slug was generated and using Ilumina HiSeq 2000 technology an approximate number of 339 millions of paired end reads was obtained. *De novo* transcript assembly performed by Trinity pipeline [9] resulted in 136,406 contigs with average lengths of 671.04 bp and N50 of 971 bp, meaning that 50% of the assembled sequences are 971 bp long or longer. 53,523 protein-coding sequences (CDS) were identified using the TransDecoder tool from the Trinity package with PFAM version 27.0. Fig 1 shows the distribution of CDS annotated contig lengths. All CDS annotated contigs of the final assembly were subjected to blastx analysis against the NCBI non-redundant protein database (nr) using the web platform Fastannotator [10] with a cut-off value of 1E-6. Duplicates and substrings were removed from the translated sequence dataset and resulting protein sequences were post-assembled using an in-house script to reduce redundancy. The final assembly contains 47,451 non-redundant protein sequences. On average 1.94 sequences of the final assembly share the same hit against the NCBI nr database (Fig 2A), due to sequencing errors and/or potential isoforms. As a result, 31,463 sequences (66.3%) were assigned to at least one protein and 15,988 (33.7%) transcripts did not have a significant hit in the NCBI nr database probably because of the lack of molecular data of mollusc species. Among 31,463 matches, only 3,380 (10.7%) were known proteins, 24,217 are predicted (77%), 3,739 are hypothetical (11.9%), and 127 are either uncharacterized, unknown or unnamed proteins (0.4%) (Fig 2B). Sequences without significant similarity to sequences present in the nr database are being referred as putative novel genes. These include taxonomically constricted genes derived from ancestral genes or appeared *de novo* from non-coding sequences [18]. The high number of transcripts lacking detectable homology with protein sequences described in the database might be also due to the high number of short transcripts (147–300 bp) in the final assembly. Of the 47,451 transcripts in the final assembly, 46.5% (22,055 transcripts) are shorter than 300 bp (Fig 1). It has been shown that non-coding RNAs [19] as well as small open reading frames are potential sources of sequences lacking detectable homology with protein sequences in nr databases. A high number of putative novel genes was identified but additional genetic as well as proteomic studies are needed to annotate them correctly and this was one of the aims of the current proteomic study described below.

**Fig 3:**
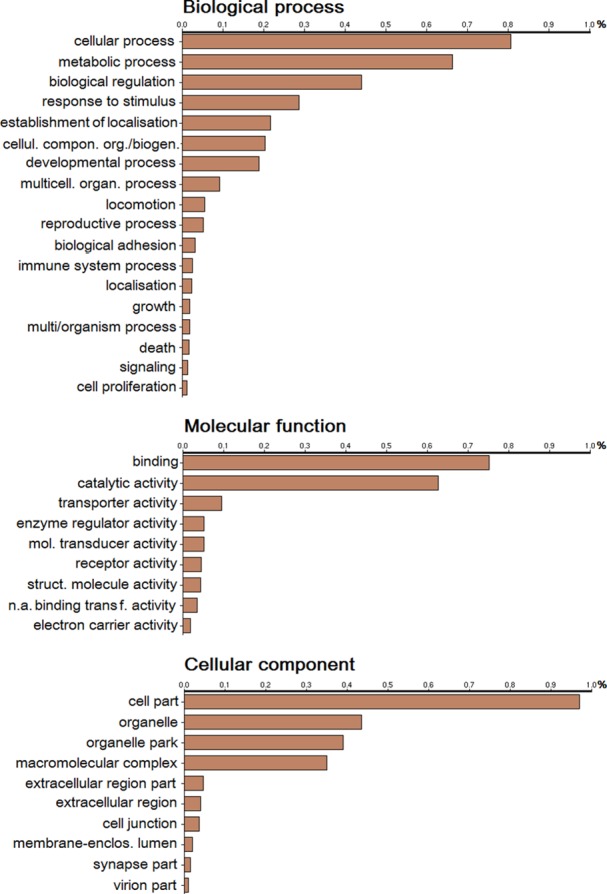
Gene Ontology (GO) analyses of the *A*. *vulgaris* transcriptome.

39% (18,561) of all sequences were functionally annotated with Gene Onthology (GO) terms using BLAST2GO tool of Fastannotator [20]. The results were summarized to the categories “biological process” (33.9%), “molecular function” (28.2%), and “cellular component” (34.8%). Within the biological process classification, metabolic processes (66%), biological regulation (43%) and response to stimulus (29%) were the most representative (Fig 3). In the molecular function category, the highest percentage of GO terms corresponded to “binding” (75%), “catalytic activity” (62%) and “transporter activity” (9.8%) (Fig 3). For the cellular component classification, the cluster sizes of “organelle/organelle part”, “macromolecular complex” and “extracellular region” were relatively large (Fig 3). Domain analysis against the Pfam database identified 16,994 of entries to have at least one domain of which 471 sequences without blastx hit were further annotated. The zinc-finger double domain zf-H2C2_2 (PF13465) was the most abundant domain followed by domain of unknown function DUF4200 (PF13863) and ankyrin repeat Ank_5 (PF13857) (S1 Table). Ankyrin domains are among the most common structural motifs in known proteins. 1,796 of transcripts were assigned with EC (Enzyme Commission) numbers.

**Fig 4:**
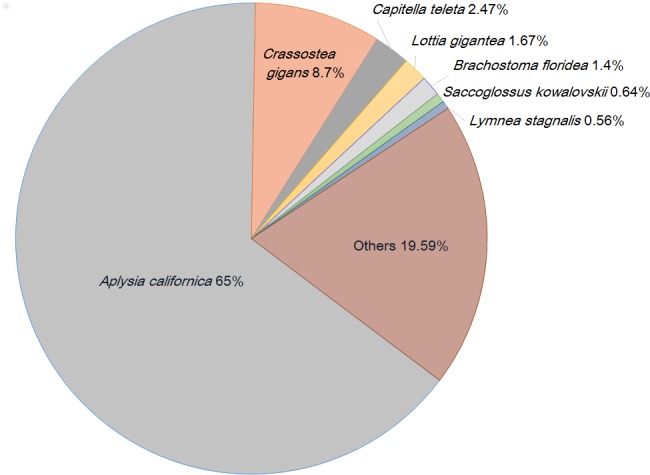
Species distribution of best blastx hits against the nr database.

We also analyzed taxonomic distribution of the best-match species of the annotable slug sequences. The highest proportion of the best blastx hits was found for *Aplysia californica* (65%), *Crassostea gigas* (8.7%) and *Capitella teleta* (2.5%) (Fig 4). Despite the limited number of molluscan sequences in the public databases, more than 80% of the sequences best matched to molluscan species. Assignment of transcripts also to chordate, plants, fungi, bacteria and viruses was probably due to more functional data on these species as well as contamination from environmental organisms during sample preparation has to be also considered.

### Proteome of slug mantle

**Fig 5:**
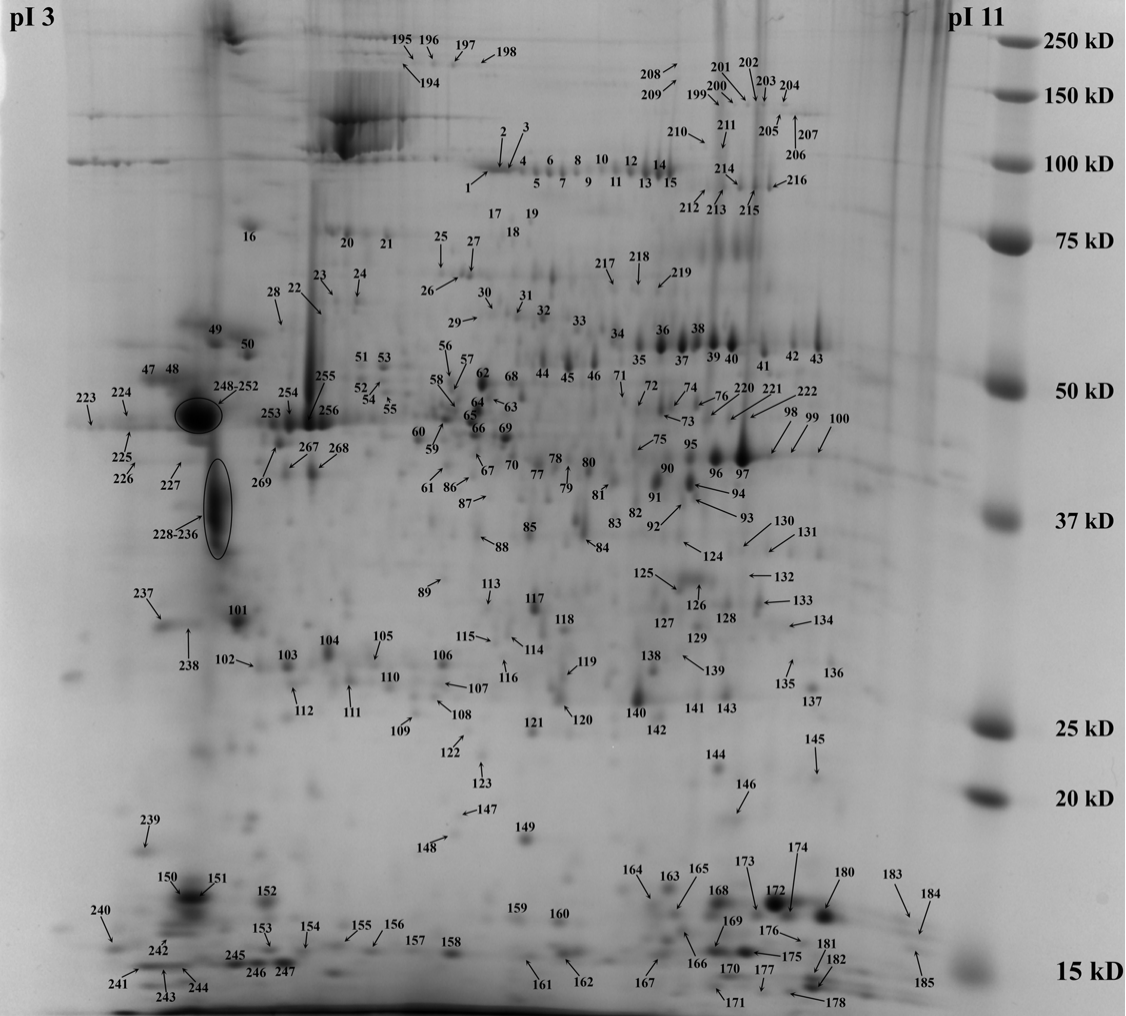
Representative 2-DE gel image from slug mantle showing protein spots selected for mass spectrometry analysis. The identified proteins are listed in S2 Table.

With the objective to gain insight into the slug proteome gel-based proteomic analysis from the *A*. *vulgaris* mantle was performed. In total, 356 spots corresponding to 833 unique proteins were clearly resolved in 2-DE reproducible gels (S2 Table). Fig 5 shows the representative 2-DE gel covering pH 3–10. Most of the spots were located between pH 5 to 9 over a broad range of MW (10–250 kDa). Among these proteins, 814 were assigned to protein sequence with significant hit in NCBI nr database and 19 represent a proteins with no significant homology in nr database. Several putative novel proteins have been identified in several spots representing isoforms with different posttranslational modifications deduced from different position in the gel. Because of the limitation of 2-DE for very small and very large proteins, alkaline proteins and hydrophobic proteins, we also analyzed the protein samples by 1-D SDS-PAGE coupled with LC−MS/MS. 2011 proteins were identified in two biological replicates from 1-D SDS-PAGE (S3 Table). Combining the results from 1-D and 2-D gel separation methods, a total of 2129 proteins were identified with minimum of one unique peptide and two distinct peptides (S4 Table). The overall false-positive assignment was estimated around 1% by a target-decoy search strategy suggesting high quality of our dataset. 1770 identified proteins (83.1%) had matches to Gene Ontology (GO) term annotations and were categorized to molecular function (1647 proteins), cellular component (1337 proteins), and biological process (1596 proteins). Of these, 23.7% have functions that are associated with cytoskeletal protein binding. The mantle, made up of muscle and skin, hence many of the cytoskeletal and muscle-related proteins were observed. The highest number of identified peptides in the combined proteomic approach were observed for paramyosine, myosine, actin and arginine kinase. Arginine kinase belongs to a class of kinases that play a crucial role in invertebrates in the maintenance of ATP levels by the phosphorylation of phosphagens which then serve as a high energy source for rapid ATP replenishment [21]. The GO terms with the largest number of assigned sequences in the biological process category were small molecule metabolic process, catabolic process, biosynthetic process and response to chemical stimulus. 1882 (88.3%) had at least one PFAM domain match and 398 (18.7%) an EC number. The RNA binding motifs RRM_6 and RRM_1 (PF14259 and PF00076) were the most abundant domains followed by Tropomyosin_1 domain (PF12718) and EF hand domains (PF13833, PF13405). (S4 Table).

Combined transcriptomic and proteomic analysis revealed the presence of a high number of sequences sharing carbohydrate-recognition domains, like lectin domains, proteins with C1q domain and toxin-like proteins were present as well (S5 Table).

## Discussion

The combined transcriptomic and proteomic approach allowed the identification of a great number of new transcripts and proteins related to metabolic, functional and cellular components. These transcriptomics and proteomics data are by far the most comprehensive among terrestrial gastropods. Moreover, one may speculate that innate immune response-related and defense elements identified in this study may contribute to uncover the success of *A*. *vulgaris* and indeed, *A*.*vulgaris* is spreading Europe-wide and is well protected against tentative predators and pathogens [22–24]. 2129 *A*. *vulgaris* mantle proteins were identified through this study, markedly expanding the list of known proteins in terrestrial gastropods, demonstrating the powerfulness of gel-based approach (2-DE and 1-D SDS-PAGE) combined with mass spectrometry identification (LC-ESI-MS/MS). Of the two protein identification approaches used in this study, the 1-D approach has shown markedly higher sensitivity as demonstrated by the significantly higher number of detected proteins. The 2-DE method is useful in separating protein isoforms with different isoelectric point and protein mobility [25].

Slugs belonging to the one of the most successful phyla, Mollusca, lack clear evidence of adaptive immunity. They contend with many pathogens, including bacteria, fungi, viruses and several lineages of specialized eukaryotic parasites, and how they without adaptive immunity effectively and sufficiently defend themselves, is not well understood. The innate immune system in gastropoda is provided by physical barriers (e.g., shell, skin and epithelium), an evolutionary archaic mechanism of molecular "self-non-self" recognition, as well as by a variety of denfense-related factors. The soft, moist slug body protected by a ciliated, mucus-producing epithelium that provides an initial physical barrier to colonization by the pathogens, plays a crucial role in host defense. The diversification of immune systems during evolution involves the expansion of particular gene families in given phyla. Analysis of transcriptomic data from *A*. *vulgaris* shows a comprehensive repertoire of genes related to innate immunity including many associated pattern recognition, effector proteins and cytokine-like proteins, such as lectins, complement-like proteins, peptidoglycan-recognition proteins (PGRPs), lipopolysaccharide and β1,3-glucan-binding proteins, fibrinogen-related proteins (FREPs), pore-forming membrane attack/perforin (MACPF) domain proteins and toxin-like proteins that indicates an extraordinary complexity of immune system with a high degree of pathogen specificity and immune-priming.

### Lectin-like proteins

Lectins play an important role in „self-non-self”recognition and clearance of invaders in gastropods. The slug repertoire of lectin-like proteins bearing conserved carbohydrate-recognition domains (CRDs) is highly diversified, including C-type lectin family, galectin, malectin, H-type lectins, L-type lectins, Ricin-B lectins and calnexin. The diversity of CRDs in lectins may reflect the different functions that the proteins perform. In total, lectin-associated functions may be attributed to 149 transcripts, 37 were unambigously identified at the protein level (S5 Table). These lectin-like proteins show low overlap between species of invertebrates, suggesting relatively rapid evolution of pattern recognition proteins involved in innate immunity. Most of transcripts/proteins described belong to tree lectin families, C-type, R-type and H-type.

### C-type lectins

**Fig 6:**
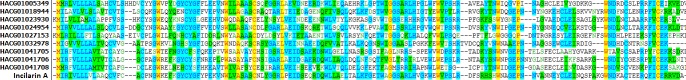
Domain architecture of C-lectin-like molecules found in the transcriptome of *A*. *vulgaris*.

**Fig 7:**
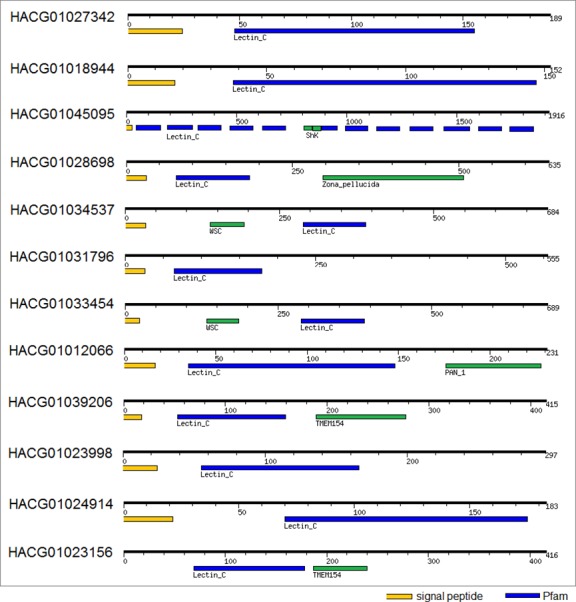
Alignment of amino acid sequences of incilarin-like proteins from *A*. *vulgaris*.

C-type lectins are a superfamily of diverse proteins able to bind specific carbohydrates in a Ca^2+^-dependent manner. The CRDs contain characteristic double-loop structures stabilized by two highly conserved disulfide bridges located at the bases of the loops [26]. The second loop is structurally and evolutionarily flexible and is involved in Ca^2+^-dependent carbohydrate binding. In total, 47 transcripts encoding proteins with C-type lectin CRDs have been found and 14 were identified also at the protein level. 17 of 21 complete transcripts have a predicted signal peptide indicating that they are secreted by cells to exert their functions (S5 Table) (Fig 6). Fig 7 shows remarkable sequence variability within incilarin-like proteins identified in our study. Incilarins are C-type lectins originaly isolated from the water-soluble fraction of the body surface mucus of the land slug, *Incilaria fruhstorfer*, possessing hemagglutination activity [27].

### R-type lectins

**Fig 8:**
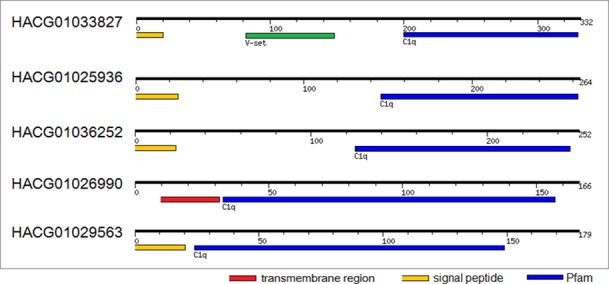
Domain architecture of galectins, H-type lectins, R-type lectins, L-type lectins and calnexin-like proteins found in the transcriptome of *A*. *vulgaris*.

The R-type lectins are members of a superfamily of proteins which contain a carbohydrate-recognition domain (CRD) structurally similar to the CRD in ricin. All identified R-type lectins, are chimeric proteins consisting of an A chain with enzymatic activity (galactosyltransferase) linked through a disulfide bridge with a B chain with lectin activity (Fig 8). An analysis of the transcriptome predicts 32 unigenes coding proteins of R-type lectins. R-type lectine-like proteins were not identified at the protein level probably due to tissue-specific expression.

### H-type lectins

**Fig 9:**
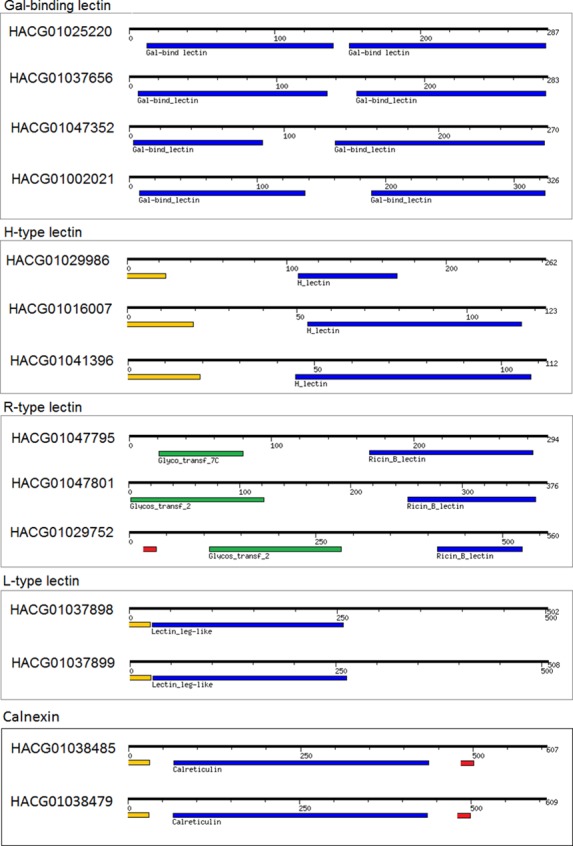
Alignment of amino acid sequences of H-type lectins from *A*. *vulgaris* with *Helix pomacea* and *Cepaea hortensis*.

The H-type lectins are proteins which contain a carbohydrate-recognition domain (CRD) structurally similar to *Helix pomatia* agglutinin (HPA) [28]. It has been shown that HPA is part of the innate immunity system of *H*. *pomatia* and a component of perivitelline fluid protecting fertilized eggs from bacteria. We identified 12 transcripts, 7 at the protein level. H-type lectins from *A*. *vulgaris* have a similar size and are predicted to share the same hexameric arrangement achieved by conserved cysteine residues involved in the intermolecular disulfide bridge formation (Figs 8 and 9).

### Galectins

Galectins represent a family of structurally-related diverse lectins with carbohydrate binding specificity primarily to β-galactoside residues. Screening of the *A*. *vulgaris* transcriptome revealed that ten unigenes coding proteins share a galectin domain. Galectin domain-containing proteins expressed in *A*. *vulgaris* are not polymorphic, only a tandem-repeat type galectin has been identified (Fig 8). The absence of a signal sequence is consistent with other galectins, which are known to be actively secreted from diverse tissue via a “nonclassical” secretory pathway [29]. All of these lectins are most similar to gastropod galectins (*Aplysia californica*, *Biomphalaria glabrata*, etc.;blastp E value ranges from 0 to 2E−32).

### L-type lectins

A member of L-type (legume-like) lectin family, ERGIC-53 is known as a pattern recognition receptor involved in the immune system of *E*. *sinensis* [30]. Transcriptomic analysis revealed the presence of two L-type lectin sequences with highest similarity to homolog of ERGIC-53 protein from *Littorina littorea* (blastp: E = 0.0; Identity = 57–58%). ERGIC-53 homologs share the characteristic architecture with an extracellular signal peptide and a single N-terminal L-type carbohydrate binding site (Fig 8).

### Calnexin

Members of the evolutionarily conserved calnexin/calreticulin-superfamily bind to oligosaccharides containing terminal glucose residues. Calnexin acts as one of the pattern recognition receptors and has a crucial role in shrimp antibacterial immunity [31]. The search of *A*. *vulgaris* transcriptomic data revealed four calreticulin-like unigenes coding calnexin proteins with a highest similarity to *Aplysia californica* homologs (blastp: E = 0.0; Identity = 70–74%). Calnexin homologs contain a signal sequence and an additional C-terminal transmembrane helix (Fig 8).

### C1q domain-containing proteins

The C1q domain-containing proteins (C1qDC) possessing lectin-like features are a family of proteins characterized by a globular C1q domain [32] regarded as an important player in innate immunity of bivalvia molluscs [33–37]. C1qDC proteins participate in several immune responses, such as pathogen recognition [38], microorganism agglutination [39] and mediating cell migration [40]. In addition, the C1q domain is involved in other immunological processes, such as phagocytosis [41], neutralization of viruses, cell adhesion and clearance of apoptotic cells. It is proposed that C1qDC proteins can activate an ancient complement system by the lectin pathway prior to the evolution of immunoglobulins [41]. The C1q domain has been considered as an extremely efficient pattern recognition domain with highly adaptive binding properties. The extreme versatility of C1q is due to the capability of the C1q domain to bind a variety of self and non-self ligands, including lipopolysaccharides [42], virus envelope proteins, outer membrane proteins from Gram-negative bacteria, phospholipids and some acute-phase proteins.

**Fig 10:**
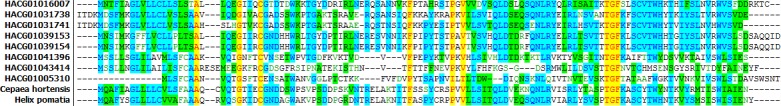
Domain architecture of C1q domain-containing proteins found in the transcriptome of *A*. *vulgaris*.

Based on sequence homology, molecular architecture and domain similarity, 34 C1q domain-containing sequences from the *A*. *vulgaris* transcriptome may be classified as members of the C1q family (S4 Table). Ten transcripts have been identified also at the protein level. Most of the complete C1qDC sequences displayed a signal peptide or a transmembrane domain on the N-terminus (Fig 10).

### Toxin-like proteins

Escaping predation is essential to survival. To reduce predation, organisms have developed a diverse defence mechanisms. A few candidate effector genes were found in the slug transcriptome, including a family of potential pore-forming membrane attack/perforin (MACPF) domain proteins. Pore-forming membrane attack/perforin (MACPF) domain proteins have been described to be involved in the biochemical defence of apple snail eggs against predators [43].

**Fig 11:**
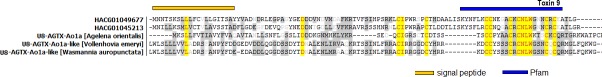
Alignment of amino acid sequences of toxin-like proteins from *A*. *vulgaris* with U8-agatoxine–Ao1a from *Agelena orientalis*.

Transcriptomic analysis of *A*. *vulgaris* revealed a novel toxin-like protein showing significant similarity to agatoxin, expressed in spider glands with a characteristic cysteine motive in the mature peptide. The agatoxin-homolog from *A*. *vulgaris* shares characteristic architecture with signal peptide, prepeptide sequence and characteristic cysteine motive in the mature peptide (Fig 11).

Taken together, one may speculate that the diversity and multitude of proteins with denfensive potential along with already reported defensins (diterpene) of this slug [44] may contribute to success and survival of this terrestrial mollusc.

### Data Accessibility

The sequence data has been submitted to the sequence read archive (SRA) database of GenBank (http://www.ncbi.nlm.nih.gov/sra) with the BioProject accession number PRJEB7891.

The mass spectrometry proteomics data have been deposited to the ProteomeXchange Consortium [45] via the PRIDE partner repository with the dataset identifier PXD002078 and 10.6019/PXD002078.

## Supporting Information

S1 Table (XLSX)

S2 Table List of 814 proteins identified from the mantel of *A*. *vulgaris* using 2-D electrophoretic separation. (XLSX)

S3 Table List of 2011 proteins identified from the mantel of *A*. *vulgaris* using 1-D electrophoretic separation. (XLSX)

S4 Table List of 2129 proteins identified from the mantel of *A*. *vulgaris*. (XLSX)

S5 Table List of lectin-like sequences and toxin-like sequences identified in *A*. *vulgaris*. (XLSX)
